# Inoculum-dependent activity of contemporary β-lactam agents against carbapenemase- producing Enterobacterales

**DOI:** 10.3389/fmicb.2026.1964408

**Published:** 2026-10-12

**Authors:** Tokeshwar Kumar Sahu, Pravin Gadkari, Jaishriram Rathored

**Affiliations:** 1 Department of Microbiology, Jawaharlal Nehru Medical College, Datta Meghe Institute of Higher Education & Research, Sawangi Meghe, Wardha, India; 2 Department of Pathology, Jawaharlal Nehru Medical College, Datta Meghe Institute of Higher Education & Research, Sawangi Meghe, Wardha, India; 3 Central Research Laboratory and Molecular Diagnostics, Jawaharlal Nehru Medical College, Datta Meghe Institute of Higher Education & Research, Sawangi Meghe, Wardha, India; 4 School of Allied Health Sciences, Datta Meghe Institute of Higher Education & Research, Sawangi Meghe, Wardha, India

**Keywords:** antimicrobial susceptibility testing, aztreonam-avibactam, carbapenemase-producing Enterobacterales, carbapenemases, cefiderocol, inoculum effect, β-lactam/β-lactamase inhibitors

## Abstract

Carbapenemase-producing Enterobacterales (CPE) pose a major therapeutic challenge because antimicrobial susceptibility measured at standard inoculum may not reflect activity at higher bacterial burdens. This Mini Review examines the biological basis, carbapenemase dependence, and agent-specific expression of inoculum effects among contemporary β-lactams. Increasing bacterial density can enhance β-lactamase burden and extracellular carbapenemase activity, alter the effective drug-to-enzyme relationship, and interact with permeability defects, bacterial physiology, and antimicrobial exposure. Available evidence shows substantial heterogeneity across carbapenemases and agents. Ceftazidime-avibactam appears comparatively stable in several CPE studies, whereas cefiderocol, aztreonam-avibactam, and other β-lactam/β-lactamase inhibitor combinations can show marked density-dependent reductions in activity under specific conditions. However, *in vitro* MIC increases do not consistently translate into reduced *in vivo* efficacy. Standardized testing approaches, mechanism-informed susceptibility assessment, and clinically relevant pharmacokinetic and pharmacodynamic studies are needed to clarify the therapeutic significance of inoculum-dependent β-lactam activity.

## 1 Introduction

Carbapenem-resistant Enterobacterales (CRE) comprise a heterogeneous group in which resistance may arise from carbapenemase production or from non-carbapenemase mechanisms, particularly the combination of β-lactamase activity with impaired outer-membrane permeability (Tamma et al., 2024). Among these organisms, carbapenemase-producing Enterobacterales (CPE) are especially important because the resistance mechanism itself influences the expected activity of newer β-lactam-based therapies (Tamma et al., 2024; Sahu et al., 2026). The therapeutic landscape has expanded with ceftazidime-avibactam, meropenem-vaborbactam, imipenem-relebactam, cefiderocol, and aztreonam-avibactam, providing important alternatives for carbapenemase-producing Enterobacterales (Barbier et al., 2023; Tamma et al., 2026). Nevertheless, susceptibility measured under routine laboratory conditions may not fully represent antibacterial activity when infection-site bacterial density exceeds that used in standard antimicrobial susceptibility testing (Lenhard and Bulman, 2019; Danjean et al., 2022).

This discrepancy is captured by the inoculum effect, in which the measured minimum inhibitory concentration (MIC) increases as the starting bacterial density increases (Lenhard and Bulman, 2019). Standard broth microdilution is performed around 5 × 10^∧^5 CFU/mL, yet recent work has shown that even variation within the accepted inoculum range can alter carbapenem susceptibility classification for some carbapenemase-producing isolates (Jaramillo Cartagena et al., 2025). In a comparative study of 40 CPE isolates tested at 10^∧^5 and 10^∧^7 CFU/mL, susceptibility-category shifts at high inoculum occurred in 88% of initially susceptible isolates for cefiderocol, 72% for imipenem-relebactam, 50% for meropenem-vaborbactam, and 8.7% for ceftazidime-avibactam (Danjean et al., 2022). These differences indicate that inoculum dependence is not uniform across contemporary β-lactams and may reflect properties of both the antibacterial agent and the underlying resistance mechanism (Danjean et al., 2022). More recently, a mechanistic study of 106 CRE isolates found a pronounced meropenem inoculum effect in carbapenemase-producing isolates but not in porin-deficient CRE, and demonstrated that released carbapenemases remained active in the extracellular milieu and could protect neighboring cells from meropenem (Jaramillo Cartagena et al., 2025).

The clinical meaning of an *in vitro* inoculum effect remains incompletely defined because infection site, bacterial burden, drug exposure, source control, and additional resistance determinants can modify treatment response (Lenhard and Bulman, 2019; Barbier et al., 2023). This uncertainty is particularly relevant for contemporary β-lactams, whose activity depends on distinct β-lactamase-inhibitor spectra, entry pathways, and pharmacodynamic properties (Barbier et al., 2023). This Mini Review therefore examines the biological basis of inoculum-dependent β-lactam activity, the contribution of specific carbapenemase mechanisms, the behavior of contemporary agents against CPE, and the major questions that remain for laboratory interpretation and clinical translation.

## 2 Literature search strategy

A focused literature search was conducted in PubMed/MEDLINE, Scopus, and Web of Science from database inception through August 2026. Search terms included combinations of “inoculum effect,” “inoculum size,” “inoculum density,” “high inoculum,” “bacterial burden,” “Enterobacterales,” “carbapenem-resistant Enterobacterales,” “carbapenemase-producing Enterobacterales,” “carbapenemase,” “KPC,” “NDM,” “VIM,” “IMP,” and “OXA-48,” together with relevant β-lactam agents, including “meropenem,” “ceftazidime-avibactam,” “meropenem-vaborbactam,” “imipenem-relebactam,” “cefiderocol,” “aztreonam-avibactam,” and “cefepime-zidebactam.” Search terms were combined using the Boolean operators AND and OR. Reference lists of relevant original articles and reviews were additionally screened to identify studies pertinent to inoculum-dependent β-lactam activity in Enterobacterales.

## 3 Biological basis of inoculum-dependent β-lactam activity

The inoculum effect does not arise from a single biological process. Instead, increasing bacterial density changes the quantitative relationship between the antimicrobial, its cellular targets, and the resistance machinery operating within the population. At a higher inoculum, the same extracellular drug concentration is distributed across a larger number of bacterial cells, reducing the effective antibiotic exposure available per cell (Aubry et al., 2025). Several density-dependent processes may reinforce this imbalance, including increased β-lactamase activity, expansion of less-susceptible subpopulations, changes in bacterial physiology, reduced expression of penicillin-binding proteins (PBPs), and biofilm- or quorum-sensing-associated responses (Lenhard and Bulman, 2019; Aubry et al., 2025).

For β-lactams, enzymatic drug degradation is one of the best-supported determinants of inoculum dependence (Lenhard and Bulman, 2019). Increasing cell density increases the overall β-lactamase burden within the bacterial population, creating greater capacity for antibiotic hydrolysis before sufficient active drug reaches its PBP targets (Lenhard and Bulman, 2019). This relationship is particularly relevant to β-lactam/β-lactamase inhibitor combinations because activity depends not only on the partner β-lactam but also on whether inhibitor exposure is sufficient relative to the amount and catalytic activity of enzyme present (Golikova et al., 2022; Kang et al., 2024). At high bacterial densities, β-lactamase activity may therefore exceed the protective capacity of an inhibitor, leaving the partner β-lactam increasingly vulnerable to hydrolysis (Lenhard and Bulman, 2019). Recent mechanistic evidence in CRE extends this concept beyond intracellular or periplasmic enzyme abundance. Carbapenemase-producing isolates released active carbapenemase into culture supernatants, and this extracellular activity protected otherwise susceptible neighboring bacteria from meropenem (Jaramillo Cartagena et al., 2025). Exposure to lethal antibiotic concentrations further increased transferable carbapenemase activity, indicating that antibiotic-mediated cell damage can expand the hydrolytic capacity of the surrounding bacterial community (Jaramillo Cartagena et al., 2025).

Inoculum dependence is also influenced by the physiological state of the bacterial population. Dense populations enter nutrient limitation and stationary-phase growth more rapidly, conditions under which β-lactam activity can decline because these agents act most effectively on actively growing cells (Lenhard and Bulman, 2019). Reduced PBP expression during stationary-phase growth has also been proposed as a contributor to diminished β-lactam activity at high inocula (Lenhard and Bulman, 2019). A larger starting population can additionally increase the absolute number of pre-existing less-susceptible cells, creating greater opportunity for survival and regrowth when antimicrobial exposure is inadequate (Strukova et al., 2023). These processes are not mutually exclusive. Their relative importance is determined by the organism, resistance background, β-lactam properties, inhibitor activity, and drug exposure, making inoculum dependence an emergent feature of the drug–bacterium interaction rather than a fixed property of an antimicrobial agent (Figure 1).

**FIGURE 1:**
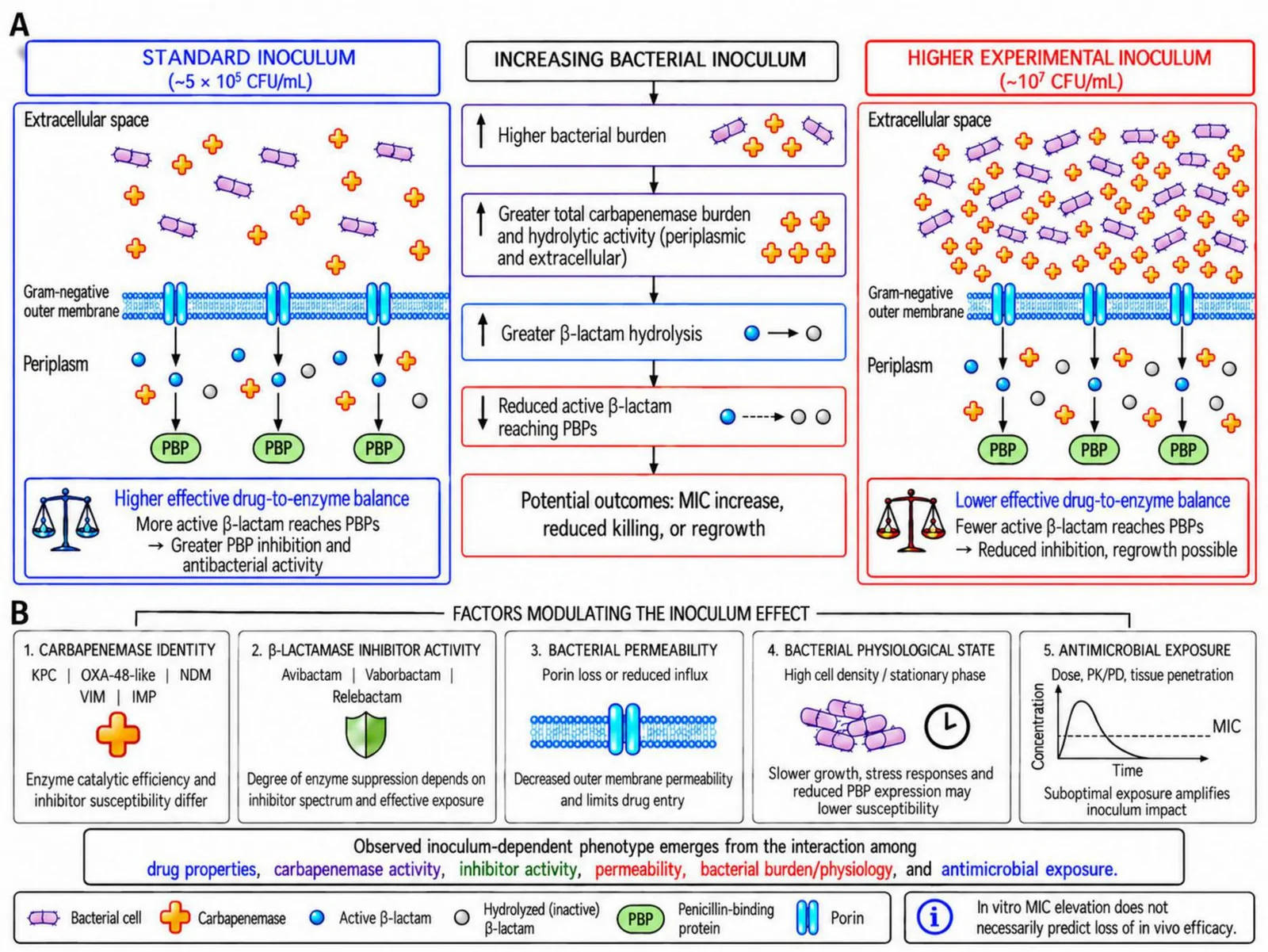
Conceptual framework of inoculum-dependent β-lactam activity in carbapenemase-producing Enterobacterales. **(A)** Comparison of standard inoculum (∼5 × 10^−7^ CFU/mL) with higher experimental inoculum (∼10^5^ CFU/mL), illustrating increased bacterial and carbapenemase burden, greater β-lactam hydrolysis, reduced active β-lactam reaching penicillin-binding proteins, and potential MIC increase, reduced killing, or regrowth. **(B)** Major factors modulating the inoculum effect, including carbapenemase identity, β-lactamase inhibitor activity, bacterial permeability, bacterial physiological state, and antimicrobial exposure.

## 4 Carbapenemase-dependent mechanisms of inoculum effects

Carbapenemase production provides a mechanistic link between bacterial density and reduced carbapenem activity, but the magnitude of this effect differs among enzyme families. In an isogenic *Escherichia coli* background, expression of seven carbapenemases representing Ambler classes A, B, C, and D produced a progressive rise in meropenem MIC as the inoculum increased, whereas expression of CTX-M-15, a non-carbapenemase β-lactamase, produced only a minimal inoculum effect (Jaramillo Cartagena et al., 2025). The same study found that NDM-1 and IMP-4 produced some of the highest meropenem resistance levels across increasing inocula, while KPC-3 produced marked resistance mainly at higher bacterial densities and OXA-48 generated comparatively lower MICs (Jaramillo Cartagena et al., 2025). These findings indicate that the inoculum effect reflects not simply the presence of a carbapenemase, but also the hydrolytic behavior of the particular enzyme against the β-lactam being tested.

For KPC-producing Enterobacterales, increasing enzyme burden can substantially compromise carbapenem activity as bacterial density rises. In a 2023 study of *Klebsiella pneumoniae*, KPC producers showed 4- to 16-fold increases in meropenem MIC at high inoculum, although the effect was less pronounced than that observed among NDM-producing isolates (Golikova et al., 2023). A subsequent study comparing KPC-2- and OXA-48/CTX-M-15-producing *K. pneumoniae* similarly detected an inoculum effect with meropenem at approximately 10^∧^7 CFU/mL, whereas ceftazidime-avibactam activity remained stable across the tested inocula (Pitart et al., 2024). This contrast suggests that effective inhibition of a susceptible serine carbapenemase can partially uncouple increasing bacterial density from loss of partner β-lactam activity.

OXA-48-like enzymes present a different phenotype because relatively modest carbapenem MICs at standard inoculum can conceal density-dependent hydrolysis. In Enterobacterales co-producing OXA-48-like enzymes and CTX-M, meropenem susceptibility decreased from 61.5% at 0.5 McFarland to 38.5% at 5 McFarland, while ceftazidime-avibactam susceptibility was preserved (Mizrahi et al., 2022). Consistent with this phenotype, OXA-48 conferred the lowest meropenem MICs among several carbapenemases expressed in an isogenic system, although a clear inoculum-dependent increase remained detectable (Jaramillo Cartagena et al., 2025).

NDM-producing Enterobacterales can show particularly pronounced meropenem inoculum effects. Among NDM-producing *K. pneumoniae*, meropenem MICs increased 8- to 256-fold at high inoculum, exceeding the shifts observed in the KPC and OXA-48 groups evaluated under the same conditions (Golikova et al., 2023). Moreover, a study of 195 CPE isolates found marked inoculum effects for both cefiderocol and aztreonam-avibactam across MBL, KPC, and OXA-48-like producers, with NDM producers showing lower cefiderocol susceptibility than IMP- or VIM-producing isolates (Huang et al., 2023).

Carbapenemase identity therefore acts together with the broader resistance background (Wilson et al., 2019; Blanco-Martín et al., 2024). Experimental disruption of OmpK36 in a KPC-producing *K. pneumoniae* increased the standard-inoculum meropenem MIC 32-fold, while introducing KPC-3 into porin-deficient isolates increased MICs by 128- to 256-fold (Jaramillo Cartagena et al., 2025). Thus, the observed inoculum effect is best interpreted as the combined consequence of carbapenemase type, enzyme burden, drug susceptibility to hydrolysis, inhibitor activity, and bacterial permeability rather than carbapenemase production alone.

## 5 Inoculum-dependent activity of contemporary β-lactam agents

Contemporary β-lactams do not respond uniformly to increasing bacterial density. In a direct comparison of 40 CPE isolates, high inoculum affected all tested agents to varying degrees, with ceftazidime-avibactam comparatively less affected than cefiderocol, imipenem-relebactam, and meropenem-vaborbactam (Danjean et al., 2022). Ceftolozane-tazobactam had limited baseline activity in the same collection (Danjean et al., 2022). These findings place bacterial density alongside carbapenemase spectrum and inhibitor potency as determinants of apparent activity.

Ceftazidime-avibactam has generally shown comparatively stable activity in CPE-focused inoculum studies, particularly against KPC- and OXA-48-like producers (Danjean et al., 2022; Mizrahi et al., 2022; Pitart et al., 2024). In OXA-48/CTX-M-producing Enterobacterales, ceftazidime-avibactam susceptibility was maintained despite twofold and tenfold increases in inoculum, while meropenem susceptibility progressively declined (Mizrahi et al., 2022). A 2024 study of KPC-2- and OXA-48/CTX-M-15-producing *K. pneumoniae* likewise detected a meropenem inoculum effect but not a ceftazidime-avibactam inoculum effect (Pitart et al., 2024). However, this relative stability is not universal. In a separate collection containing ESBL-, AmpC-, and KPC-producing *E. coli* and *K. pneumoniae*, an eightfold or greater MIC increase between 10^∧^5 and 10^∧^7 CFU/mL occurred in 52.6% of isolates with ceftazidime-avibactam and 26.3% with imipenem-relebactam (Wang et al., 2023). Increasing avibactam or relebactam from 4 to 8 mg/L reduced the frequency of these inoculum effects, supporting the importance of inhibitor exposure at high bacterial density (Wang et al., 2023). Cefepime-zidebactam also showed marked inoculum dependence against MBL-producing Enterobacterales, with MICs increasing ≥32-fold across the tested inoculum range, whereas the combination was much less inoculum dependent against non-MBL producers, including OXA-48-producing isolates (Mushtaq et al., 2022).

Cefiderocol is distinctive because its activity extends across serine carbapenemase- and metallo-β-lactamase-producing Enterobacterales, yet its *in vitro* activity can be strongly inoculum dependent (Huang et al., 2023; Sadek et al., 2023; Santerre Henriksen et al., 2024). Among KPC variants selected for ceftazidime-avibactam resistance, increasing the inoculum to 10^∧^7 CFU/mL caused large cefiderocol MIC increases and rendered all tested isolates resistant (Hobson et al., 2021). A larger study of 195 CPE found cefiderocol inoculum effects in 95.9% of susceptible isolates, with 83.6% shifting from susceptible to resistant at high inoculum (Huang et al., 2023). NDM producers also showed lower baseline cefiderocol susceptibility than IMP- or VIM-producing isolates in that collection (Huang et al., 2023).

Aztreonam-avibactam has emerged as an important treatment option for MBL-producing Enterobacterales and is currently recommended, together with cefiderocol, as a preferred option for NDM-producing Enterobacterales (Tamma et al., 2026). Within the carbapenemase-producing subgroup, the inoculum effect occurred in 42.9% of isolates with aztreonam-avibactam and 9.1% of evaluable isolates with ceftazidime-avibactam (Kim et al., 2020). In the 195-isolate CPE study, among isolates with an aztreonam-avibactam MIC ≤ 4/4 mg/L at standard inoculum, 95.2% showed an inoculum effect and 94.7% crossed the study-defined susceptibility threshold at high inoculum (Huang et al., 2023). This signal has also been reproduced *in vivo*: in a murine peritonitis model caused by CTX-M-15/NDM-1-producing *E. coli*, increasing the inoculum markedly reduced aztreonam-avibactam activity, while the *in vitro* MIC rose to 64 mg/L at 10^∧^7 CFU/mL (Amoura et al., 2025).

Taken together, available evidence does not identify any contemporary β-lactam as consistently inoculum-independent across CPE backgrounds (Danjean et al., 2022; Huang et al., 2023; Wang et al., 2023). Ceftazidime-avibactam appears comparatively resilient in several CPE datasets, whereas cefiderocol and aztreonam-avibactam can show striking density-dependent MIC shifts despite broad baseline activity. The clinically relevant phenotype therefore depends on the specific drug–inhibitor pair, carbapenemase background, bacterial burden, and achievable exposure rather than standard-inoculum susceptibility alone. The principal experimental studies evaluating inoculum-dependent activity of contemporary β-lactams against carbapenemase-producing Enterobacterales are summarized in Table 1.

**TABLE 1:** Key experimental studies informing inoculum-dependent β-lactam activity in carbapenemase-producing Enterobacterales.

References	Isolate characteristics	β-lactamase profile	Antimicrobial agent(s)	Inoculum conditions and methodology	Inoculum-effect assessment	Findings
Kim et al., 2020	81 CRE; CP-CRE subgroup, *n* = 35	KPC, NDM, other carbapenemases	CZA; ATM-AVI	10^∧^5 vs. 10^∧^7 CFU/mL; BMD	≥8-fold MIC increase	CP-CRE IE: CZA 9.1%; ATM-AVI 42.9%
Mizrahi et al., 2022	39 ceftazidime-resistant Enterobacterales isolates	OXA-48/OXA-181 + CTX-M	CZA; meropenem	0.5, 1 and 5 McFarland; gradient diffusion	MIC and susceptibility-category changes	CZA remained stable; meropenem susceptibility fell 61.5%→38.5%
Danjean et al., 2022	40 clinical CPE isolates	KPC, OXA-48-like, VIM, NDM, GES	Cefiderocol; C/T; CZA; IMR; MVB	10^∧^5 vs. 10^∧^7 CFU/mL; BMD	≥8-fold MIC increase and susceptibility-category change	Category shifts: cefiderocol 88%; C/T 75%; IMR 72%; MVB 50%; CZA 8.7%.
Huang et al., 2023	195 clinical CPE isolates	NDM (*n* = 74), IMP (*n* = 42), VIM (*n* = 27), KPC (*n* = 38), OXA-48-like (*n* = 14)	Cefiderocol; ATM-AVI	10^∧^5 vs. 10^∧^7 CFU/mL; BMD	≥8-fold MIC increase and study-defined threshold shift	IE: cefiderocol 95.9%; ATM-AVI 95.2%
Wang et al., 2023	19 *Escherichia coli* and *Klebsiella pneumoniae* isolates; 16 clinical and 3 standard isolates	ESBL, KPC and AmpC producers	CZA; IMR	10^∧^5 vs. 10^∧^7 CFU/mL; BMD	≥8-fold MIC increase	IE: CZA 52.6% (10/19); IMR 26.3% (5/19)
Pitart et al., 2024	20 *Klebsiella pneumoniae* isolates	OXA-48 + CTX-M-15; KPC-2; CTX-M-15-only comparator background	Meropenem; CZA	1–5 × 10^∧^5 vs. 1–5 × 10^∧^7 CFU/mL; BMD	MIC-based inoculum response	IE with meropenem; not detected with CZA
Godron et al., 2024	Isogenic *Escherichia coli* parental and NDM-1-producing strains; *in vitro* and murine peritonitis models	NDM-1	Cefiderocol	*In vitro*: 10^∧^5 vs. 10^∧^7; *in vivo*: 10^∧^6 vs. 10^∧^8	MIC, bactericidal activity, survival, organ sterilization and bacterial burden	MIC 2→1024 mg/L; strong *in vitro* IE not reproduced *in vivo*
Jaramillo Cartagena et al., 2025	106 clinical CRE isolates across 6 genera and 12 species; mechanistic experiments in isogenic *E. coli*	KPC, NDM, IMP, VIM, OXA-48, SME; porin-deficient CRE comparators	Meropenem	Broad-range BMD; IE quantified at 4 × 10^5^ vs. 1.3 × 10^7^ CFU/mL	High-/standard-inoculum MIC ratio; ≥8-fold MIC increase also evaluated	All 36 CP-CRE showed a clear IE, whereas porin-deficient CRE showed none; extracellular carbapenemase activity mediated transferable protection
Amoura et al., 2025	Isogenic *E. coli*; *in vitro* + murine model	NDM-1; CTX-M-15 + NDM-1	ATM-AVI	*In vitro*: 10^∧^5 vs. 10^∧^7; *in vivo*: 10^∧^6 vs. 10^∧^8 CFU	MIC, bacterial killing and *in vivo* efficacy	ATM-AVI MIC reached 64 mg/L; high inoculum reduced *in vivo* activity

ATM-AVI, aztreonam-avibactam; BMD, broth microdilution; C/T, ceftolozane-tazobactam; CPE, carbapenemase-producing Enterobacterales; CP-CRE, carbapenemase-producing carbapenem-resistant Enterobacterales; CRE, carbapenem-resistant Enterobacterales; CZA, ceftazidime-avibactam; IE, inoculum effect; IMR, imipenem-relebactam; MIC, minimum inhibitory concentration; MVB, meropenem-vaborbactam.

## 6 Current gaps and future directions

Despite increasingly consistent evidence that bacterial density can alter β-lactam activity, the field still lacks a standardized framework for measuring and interpreting the inoculum effect. Contemporary studies have used different inoculum ranges and endpoints, including categorical susceptibility shifts, fold changes in MIC, and dynamic killing models (Danjean et al., 2022; Aubry et al., 2025). Danjean et al. (2022) compared 10^∧^5 with 10^∧^7 CFU/mL and focused on susceptibility-category changes, whereas Aubry et al. (2025) modeled bacterial killing across four inocula from 10^∧^4 to 10^∧^8 CFU/mL. This methodological heterogeneity limits direct comparison across agents and carbapenemase backgrounds. Future studies should therefore report inoculum size, growth conditions, inhibitor concentration, carbapenemase genotype, permeability defects, MIC shift, and categorical change using harmonized definitions.

The most important unresolved question is whether an *in vitro* inoculum effect predicts loss of efficacy *in vivo*. For cefiderocol, an NDM-1-producing *Escherichia coli* showed a striking increase in MIC at high inoculum *in vitro*, yet increasing the inoculum did not significantly alter survival, organ sterilization, or bacterial counts in the NDM-1 model of murine peritonitis (Godron et al., 2024). In contrast, high inoculum markedly reduced the *in vivo* activity of aztreonam-avibactam against CTX-M-15/NDM-1-producing *E. coli* in another murine peritonitis model (Amoura et al., 2025). These divergent findings show that an MIC shift alone cannot be assumed to represent clinical failure (Figure 1). Experimental models should therefore incorporate infection-site bacterial burden, host immunity, source control, and clinically achievable drug exposure, followed by prospective clinical studies linking these variables with microbiological and clinical outcomes.

Pharmacokinetic/pharmacodynamic integration is another priority (Gill et al., 2021; Kunz Coyne et al., 2025). In a KPC-3-producing *Klebsiella pneumoniae* model, ceftazidime-avibactam and colistin displayed inoculum-dependent pharmacodynamic changes, with regrowth at intermediate-to-high inocula and failure to eradicate bacteria at 10^∧^8 CFU/mL (Aubry et al., 2025). Such approaches could define whether altered dosing, prolonged exposure, or combination therapy can compensate for density-dependent loss of activity.

Finally, the relevance of inoculum variation to routine susceptibility testing requires careful validation. Recent work showed that 50% and 24% of carbapenemase-producing CRE changed meropenem and ertapenem susceptibility classifications, respectively, across the inoculum range permitted in clinical testing (Jaramillo Cartagena et al., 2025). Before high-inoculum testing can be considered for routine use, reproducible, drug-specific thresholds that correlate with treatment outcomes will be required.

## 7 Conclusion

The inoculum effect is an important but incompletely resolved determinant of β-lactam activity against carbapenemase-producing Enterobacterales. Available evidence shows that increasing bacterial density can substantially alter MICs and antibacterial activity, although the magnitude of this response varies across β-lactams, carbapenemase backgrounds, and experimental conditions. These differences are best understood as the result of interacting determinants, including carbapenemase identity and abundance, inhibitor activity, bacterial permeability, physiological state, and effective antimicrobial exposure. Contemporary agents therefore cannot be classified simply as inoculum-dependent or inoculum-independent. Although ceftazidime-avibactam appears comparatively stable in several CPE datasets, marked density-dependent changes have been reported for cefiderocol, aztreonam-avibactam, and other β-lactam/β-lactamase inhibitor combinations under specific conditions. Importantly, large *in vitro* MIC shifts do not consistently predict reduced *in vivo* efficacy, emphasizing the need for cautious clinical interpretation. Standardized inoculum-effect definitions, mechanism-informed susceptibility studies, and pharmacokinetic/pharmacodynamic models linked to clinically relevant bacterial burdens are needed to determine when inoculum-dependent activity should influence therapeutic decision-making and laboratory interpretation. Until such evidence is available, high-inoculum findings should be regarded as mechanistic and translational signals rather than direct predictors of treatment failure.
